# The Application of Fluorescence Optical Imaging in Systemic Sclerosis

**DOI:** 10.1155/2015/658710

**Published:** 2015-08-18

**Authors:** Alexander Pfeil, Karl F. Drummer, Joachim Böttcher, Christian Jung, Peter Oelzner, Diane M. Renz, Marcus Franz, Andreas Hansch, Gunter Wolf

**Affiliations:** ^1^Department of Internal Medicine III, Jena University Hospital, Friedrich Schiller University Jena, Erlanger Allee 101, 07747 Jena, Germany; ^2^Institute of Diagnostic and Interventional Radiology, SRH Wald-Klinikum Gera, Straße des Friedens 122, 07548 Gera, Germany; ^3^Department of Internal Medicine I, Jena University Hospital, Friedrich Schiller University Jena, Erlanger Allee 101, 07747 Jena, Germany; ^4^Department of Radiology, Charité-University Medicine Berlin, Charitéplatz 1, 10117 Berlin, Germany; ^5^Institute of Diagnostic and Interventional Radiology, Heinrich Braun Clinic Zwickau, Karl-Keil-Straße 35, 08060 Zwickau, Germany

## Abstract

*Objective*. The aim of this study was to visualize soft tissue inflammation using FOI on patients with Systemic Sclerosis (SSc) characterized by SSc-related Raynaud's phenomenon and to detect the therapeutic response to treatment with iloprost or alprostadil.* Methods*. Twenty-one patients with SSc and Raynaud's phenomenon and twenty-six healthy controls were prospectively included. The SSc patients were intravenously treated with iloprost or alprostadil over seven days. FOI was performed at baseline and after seven days using an intravenous application of indocyanine green (ICG). The hands were divided into nineteen segments per hand. All segments were quantitatively evaluated to determine changes in ICG.* Results*. The sensitivity and specificity of FOI in the detection of ICG enhancement in patients with SSc were 95% versus 96%. At baseline, 31.5% hand segments showed ICG enhancement. After seven days of either iloprost or alprostadil therapy a significant reduction in the ICG was observed which ranged from 40.9% to 24.7%.* Conclusion*. The study demonstrates that the FOI technique is able to visualize soft-tissue inflammation with both high sensitivity and specificity. The anti-inflammatory therapeutic effects of iloprost were slightly stronger than alprostadil. FOI offers promising benefits in the diagnosis and therapy of patients with SSc-associated Raynaud's phenomenon.

## 1. Introduction

Systemic Sclerosis (SSc) is a connective tissue disease affecting various organs including the peripheral vessels [1, 2]. A characteristic clinical feature of the disease is peripheral vessel involvement associated with Raynaud's phenomenon and digital ulceration [2]. The multifactorial genesis of SSc is complex and three types of cells are mainly involved in the immunological process: fibroblasts, endothelial cells, and cells of the immune system (in particular mononuclear cells, T-lymphocytes, and B-lymphocytes) [3]. Mononuclear cells and T-cells produce inflammatory cytokines and growth factors which are associated with the onset and progression of fibrosis and induce autoinflammatory microvascular damage [4]. Such microvascular damage is characteristically predominant in SSc [1] leading to Raynaud's phenomenon, a vasospastic disorder of the fingers [5].

For treatment of Raynaud's phenomenon the vasodilator prostaglandin I2 analogue iloprost [6] and the prostaglandin E1 analogue alprostadil are widely used [7].

Fluorescence optical imaging (FOI) is in vivo technique to visualize inflammation based on the accumulation of fluorescence optical contrast media (e.g., indocyanine green) in inflammatory-altered tissue. Several in vitro and in vivo studies have revealed the potential of FOI to detect inflammatory arthritis [8–11]. Pfeil at al. were the first to describe the use of FOI as in vivo technique for the visualization of inflammation in the hands based on the accumulation of fluorescence optical contrast media in patients with SSc-associated Raynaud's phenomenon [12].

The aim of this prospective study was to verify the sensitivity and specificity of FOI in the detection of SSc-associated soft tissue inflammation. The visualization of inflammation before and after treatment with iloprost or alprostadil using FOI in patients with SSc-associated Raynaud's phenomenon was performed and subsequently compared to healthy controls.

## 2. Methods

### 2.1. Patients

The study comprised 47 participants (26 healthy controls and 21 SSc patients).

#### 2.1.1. Healthy Control Group

The control group consists of 26 healthy participants (11 men and 15 women) without any clinical signs of inflammatory joint disease or soft tissue disease. The mean age was 24.8 ± 3.3 years.

#### 2.1.2. Patients with Systemic Sclerosis

The prospective longitudinal study includes twenty-one patients (1 male and 20 females) with SSc following criteria set by the American College of Rheumatology [13]. All patients presented typical Raynaud's phenomenon. The following exclusion criteria were defined:

- Contrast media allergy (e.g., ICG).
- Recent traumatic injury of the hands.
- Swollen joints of the hands or fingers.

At baseline the mean age was 63.7 ± 8.7 years and mean disease duration was 12.1 ± 10.0 years. 33.3% of the patients were positive for Antinuclear Antibodies (ANA), 4.8% were Antitopoisomerase I (anti-SCL70) positive, 14.3% were Anticentromere Antibodies (ACA) positive, and 33.3% were positive for ANA as well as anti-SCL70. The mean C-reactive protein was 3.2 ± 1.8 mg/L. Additionally, 9 patients were treated with calcium channel blockers which showed no influence on the below-mentioned data. No patients received nonsteroidal anti-inflammatory drugs or cox inhibitors. Clinical characteristics of the patients are detailed in Table 1.

The patients underwent continuous intravenous infusion of iloprost (20 *μ*g, Ilomedin, *n* = 16) or alprostadil (60 *μ*g, Prostavasin, *n* = 5) via peripheral venous catheter (20 and 22 gauges) in an arm vein using a standard protocol [5]. The duration of infusion was six hours for seven consecutive days.

### 2.2. Methods

FOI (Xiralite system, Mivenion GmbH, Berlin, Germany) visualizes hand perfusion by detection of fluorescence signals emitted by ICG. The ICG excites light emitting diodes and the fluorescent signals are detected with a sensitive camera system [11]. The camera is set up in a computerized system with specialized software. The computer records the acquired FOI images with a standard frame rate of one image per second over a standard period of six minutes [11]. In total 360 images were acquired for each examination [11].

In total 360 images were acquired for each examination [11], one image per second.

The FOI image acquisition was performed by a standardized examination procedure:

- Both hands were placed in the scanner.
- Indocyanine green (ICG-Pulsion, 0.1 mg/kg bodyweight) was intravenously injected via a peripheral venous catheter (20 and 22 gauges) in an arm vein ten seconds after the start of the examination.
- The FOI acquired one image per second during an examination period of 360 seconds.
- FOI was performed before (baseline) and after the seventh day of iloprost or alprostadil infusion.
- All FOI examinations were performed in the same room under a stable room temperature.

All images were analyzed using Xiralite software (XiraView Software, version 3.6). By adapting the study protocol of Werner et al., the acquired image sequences for each examination (total 360 images per patient) were divided into three phases:* Phase 1 included* all images from the start of the FOI examination to visible ICG signals in the fingers.* Phase 2 included* all images with an intensity maximum at the hand and fingers.* Phase 3 included* all images from the end of phase 2 to the end of the FOI examination. For the interpretation of the FOI examinations, the images of phase 2 with the peak ICG enhancement at the hands and fingers were used for the scoring [14].

### 2.3. Scoring of the Fluorescence Optical Images

**Figure 1:**
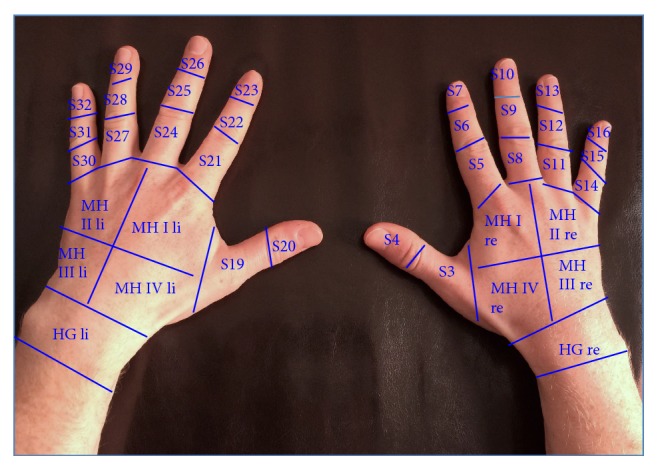
Segments of both hands for quantification of inflammatory associated enhancement of indocyanine green in SSc (S = segment, MH = middle hand).

All images were read in consensus by three observers to determine the ICG enhancement of both hands. Each hand was divided into 19 segments (see Figure 1). All 38 segments from both hands were analyzed and ICG fluorescence enhancement was determined per hand, and the total sum score from both hand segments was archived.

### 2.4. Reliability of the Fluorescence Optical Scoring

The reliability of the FOI scoring was evaluated for interrater reliability and intrarater reliability. Intrarater reliability evaluated the reliability of the FOI scoring for one reader and the interrater reliability showed the reliability for two different readers.

### 2.5. Ethics

The study protocol was approved by the Ethics Committee of the Friedrich Schiller University Jena. All study participants signed consent forms after receiving written and oral information.

### 2.6. Statistical Analysis

The statistical analysis consists of the following steps:

(i) The interrater reliability and intrarater reliability were quantified based on the kappa value. The *κ* coefficients of agreement between the observers were determined according to the Fleiss classification (poor < 0.40; moderate 0.40–0.59; good 0.60–0.75; excellent > 0.75) [15].
(ii) Sensitivity and specificity of FOI in the detection of SSc-associated ICG enhancement were verified by ROC-curve analysis between healthy controls and patients with SSc.
(iii) The statistical analysis focused on the differences of the enhancement of ICG before (baseline) and after iloprost or alprostadil therapy. The primary endpoint of the study was the ICG enhancement as detected by FOI at day 7 after iloprost or alprostadil application. The differences for both treatment groups between the two time points were detected by the paired *t*-test.

The significance level was *p* < 0.05. Statistical analysis was performed using SPSS version 15.0 (SPSS, Chicago, Illinois, USA), for Windows, respectively.

## 3. Results

### 3.1. Reliability

The kappa coefficient of the interrater reliability was 0.90 (*p* < 0.001). For the intrarater reliability a higher kappa coefficient was observed (*κ* coefficient 0.95, *p* < 0.001).

### 3.2. Sensitivity and Specificity

The sensitivity and specificity of FOI in the detection of SSc-associated ICG enhancement were 95% versus 96% with an accuracy of 97%. The positive predictive value was 95% and the negative predictive value revealed 96%.

### 3.3. Description of Indocyanine Green Enhancement at Baseline

The patients with SSc presented a plane enhancement of ICG in both hands. In total 798 hand segments in SSc were scored, where 251 hand segments (31.5%) revealed an enhancement of ICG. The highest enhancement of ICG was observed in the segments of the proximal phalanges I–V (right hand: 49 segments, left hand: 54 segments). Additionally the middle hand (right hand: 21 segments, left hand: 27 segments) as well as the end phalanges I–V (right hand: 21 segments, left hand: 26 segments) of both hands revealed ICG enhancement before the therapy of iloprost or alprostadil was initiated (Figure 3).

### 3.4. The Course of Indocyanine Green Enhancement after Therapy with Iloprost or Alprostadil

**Figure 2:**
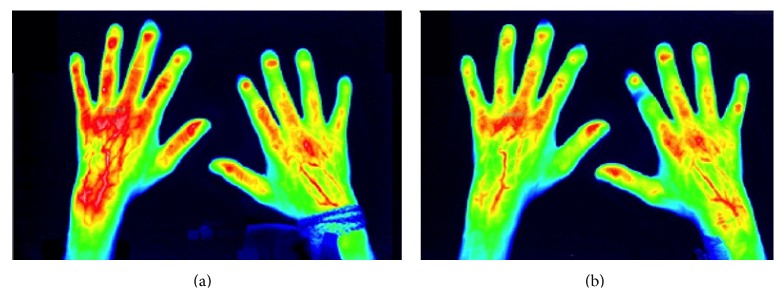
(a) Initial imaging of the enhancement of indocyanine green by fluorescence optical imaging as a marker of inflammatory activity in patients with SSc and associated Raynaud's phenomenon before the application of alprostadil (day 0, baseline) and (b) after the alprostadil therapy (day seven), corresponding to the reduced enhancement of indocyanine green after the iloprost therapy over 7 days [12].

**Figure 3:**
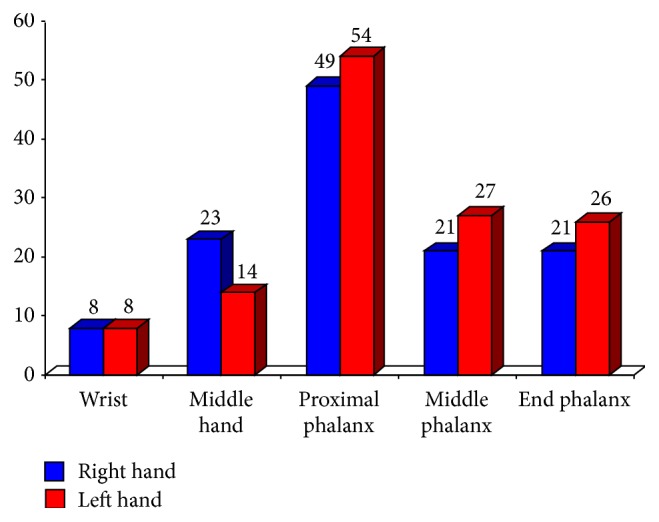
Distribution of indocyanine green (ICG) enhancement in different segments of both hands at baseline (*n* = 21).

All patients were treated with intravenous application of iloprost or alprostadil over seven consecutive days. By comparing the ICG enhancement at day 0 and after the application of iloprost over seven days, a significantly (*p* < 0.05) reduced enhancement of ICG in 166 versus 98 hand segments at the end of therapy was observed. A reduction of −40.9% (*p* < 0.05) in ICG enhancement between both time points was observed using iloprost. Regarding the alprostadil group a decrease of ICG enhancement with −24.7% between baseline (85 hand segments) and day 7 (64 hand segments) was verified (see Table 2 and Figure 2).

## 4. Discussion

SSc is a systemic autoimmune disease associated with inflammatory alterations and also Raynaud's phenomenon. To the best of our knowledge, this is the first study which illustrates the inflammatory changes in patients with SSc and associated Raynaud's phenomenon in an investigation using in vivo fluorescence optical imaging to determine the differentiation in therapeutic effects between iloprost or alprostadil compared to healthy subjects.

### 4.1. Indocyanine Green Enhancement in SSc with Raynaud's Phenomenon

Using FOI an accumulation of ICG was observed in both hands before iloprost or alprostadil therapy with high sensitivity and specificity (sensitivity 95%, specificity 96%, and accuracy 97%) compared to healthy subjects. Generally, FOI detected ICG enhancement in the inflamed tissue [10] which showed a plane ICG enhancement of both hands and fingers in SSc with Raynaud's phenomenon. The enhancement of fluorescence optical contrast media was observed in vitro in the inflammatory tissue as visualized by FOI with an excellent correlation to histopathology. The accumulation of fluorescence optical contrast media has been discussed as an inflammatory-related vascular leakage and an increased permeability for macroglobulins [9].

Furthermore, cytokines are associated with a diffuse endothelial damage and an increased capillary permeability in patients with SSc [16]. Bollinger and colleagues demonstrated an elevated diffusion of intravenous dye with significantly enhanced fluorescent light intensities in the juxtacapillary region as detected by dynamic fluorescence videomicroscopy [17].

### 4.2. Therapeutic Effects as Detected by Fluorescence Optical Imaging

Marasini et al. reported on the clinical benefits and efficacy of iloprost or alprostadil therapy in patients with connective tissue disease and associated Raynaud's phenomenon [18]. Yet studies comparing the pharmacological properties, therapeutic effects, and immunomodulating effects of iloprost and alprostadil remain limited. This study observed a reduction (iloprost −40.9%; alprostadil −24.7%) of indocyanine green enhancement after intravenous treatment using iloprost or alprostadil.

The decreased indocyanine green enhancement getting repetitive after this vasodilator treatment is associated with a reduced inflammatory activity of SSc in the hands and fingers. In vitro and in vivo studies have already shown the anti-inflammatory and immunomodulating effects of iloprost as prostaglandin I2 analogue [19, 20]. In SSc, T-lymphocytes play a key role in the autoinflammatory process [3] showing an accentuated activation and an increase in the circulation of T-lymphocytes [21, 22]. The increases of circulating T-lymphocytes are related to high levels of inflammatory mediators [23, 24], in particular the tumor necrosis factor *α* (TNF *α*) [19, 25]. In autoimmune disease TNF *α* is the main target for monoclonal antibodies (e.g., rheumatoid arthritis) [26]. The application of iloprost reduces the TNF *α*-producing T-lymphocytes in the peripheral blood [4, 19, 20, 25]. Furthermore, the study of D'Amelio et al. observed a reduced ability of TNF *α*-positive T-lymphocytes to secrete TNF *α* after antigenic stimulus [4]. These facts highlight the anti-inflammatory effects of iloprost. Consequently, the therapeutic use of iloprost may be useful as a vasodilator and immunomodulatory agent in patients with SSc-related Raynaud's phenomenon [4].

## 5. Conclusion

In vivo fluorescence optical imaging is a technique for the imaging of inflammation in patients suffering from SSc and related Raynaud's phenomenon. Used in a novel way, the FOI can also visualize the inflammation of soft tissue and peripheral vessels of the hands and fingers at baseline. After iloprost or alprostadil therapy a remarkable reduction in the soft tissue inflammation was observed by FOI. The FOI may be a promising technique for the visualizing of autoinflammatory alterations not only for diagnostic purposes, but also for the diagnosis as well as monitoring of patients with SSc-related Raynaud's phenomenon.

## Figures and Tables

**Table 1:** Characterization of the study cohort.

	Total study cohort	Iloprost group	Alprostadil group
Patients	*n* = 21	*n* = 16	*n* = 5
Women	*n* = 20	*n* = 15	*n* = 5
Men	*n* = 1	*n* = 1	*n* = 0
Mean age and standard deviation in years	63.7 ± 8.7	64.4 ± 7.6	61.4 ± 12.4
Disease duration and standard deviation in years	12.1 ± 10.0	10.4 ± 8.0	17.6 ± 14.4
Laboratory findings			
C-reactive protein (mean and standard deviation)	3.2 ± 1.8 mg/L	3.3 ± 2.1 mg/L	2.9 ± 1.3 mg/L
Immunological findings			
Antinuclear Antibodies (ANA) positive	*n* = 7 (33.3%)	*n* = 5 (31.3%)	*n* = 2 (40.0%)
Antitopoisomerase I (anti-SCL70) positive	*n* = 1 (4.8%)	*n* = 1 (6.3%)	*n* = 0 (0%)
ANA and anti-SCL70 positive	*n* = 7 (33.3%)	*n* = 6 (37.5%)	*n* = 1 (20.0%)
Anticentromere Antibodies (ACA) positive	*n* = 3 (14.3%)	*n* = 2 (12.5%)	*n* = 1 (20.0%)
Comedication			
Calcium channel blockers	*n* = 9 (42.8%)	*n* = 6 (37.5%)	*n* = 3 (60.0%)
Immunosuppressive agents			
Mycophenolate-mofetil	*n* = 4 (19.0%)	*n* = 4 (25.0%)	*n* = 0 (0%)
Azathioprine	*n* = 9 (42.8%)	*n* = 7 (43.8%)	*n* = 2 (43.8%)
Leflunomide	*n* = 1 (4.8%)	*n* = 1 (6.3%)	*n* = 0 (0%)

**Table 2:** Levels of ICG enhancement before (day 0, baseline) and after (day 7) therapy with iloprost or alprostadil.

	Iloprost group	Alprostadil group	Total group
	(*n* = 16)	(*n* = 5)	(*n* = 21)
	Total hand segments	Total hand segments	Total hand segments
	Mean ± SD	Mean ± SD	Mean ± SD
Day 0 (baseline)	166 10.4 ± 7.4	85 17.0 ± 9.8	251 12.0 ± 8.3

Day 7	98 6.1 ± 4.9	64 12.8 ± 6.8	162 7.7 ± 5.9

*Relative reduction in %* Significance	40.9% *p* < 0.05	24.7% *p* < 0.05	35.5% *p* < 0.05

Note: SD: standard deviation.

## Abbreviations

ACA: Anticentromere Antibodies
ANA: Antinuclear Antibodies
Anti-SCL70: Antitopoisomerase I
FOI: Fluorescence optical imaging
ICG: Indocyanine green
S: Segment
SSc: Systemic Sclerosis
TNF *α*: Tumor necrosis factor *α*.
